# Supplementary material for: The interactome of intact mitochondria by cross-linking mass spectrometry provides evidence for coexisting respiratory supercomplexes
Source: Mol Cell Proteomics. 2017 Dec 8;17(2):216–32. doi: 10.1074/mcp.RA117.000470 (PMC5795388; doi:10.1074/mcp.RA117.000470)
Supplement: Supplemental Data [file supp_RA117.000470_133922_0_supp_23978_fzffwf.zip › spectra_annotation/mito_DR_spectra_annotation/135-1-12-1-5-1.pdf]

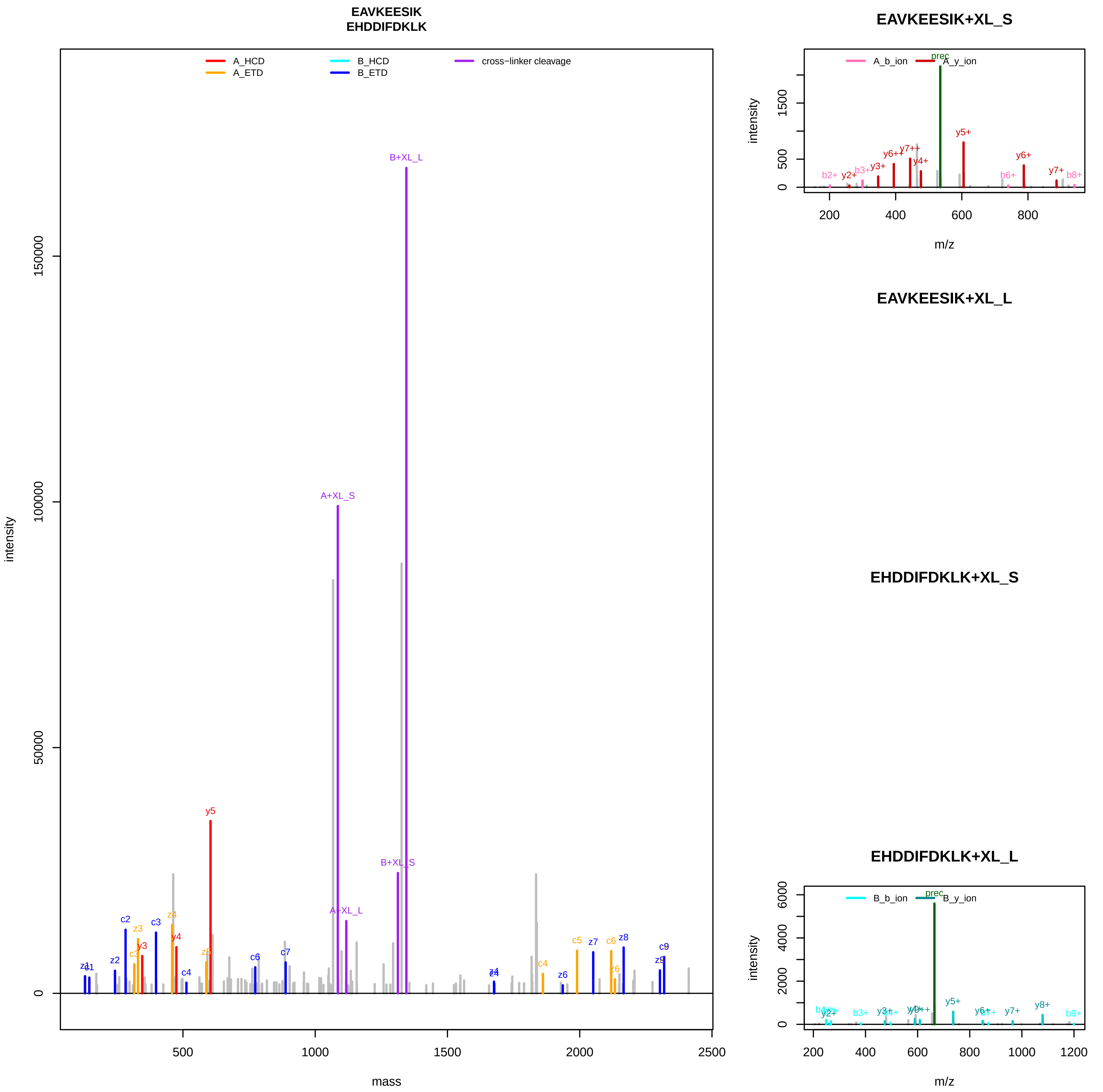

EAVKEESIK
EHDDIFDKLK
A_HCD
A_ETD
B_HCD
B_ETD
cross−linker cleavage
B+XL_L
A+XL_S
B+XL_S
A+XL_L
y5
y4
y3
z4
z3
c3
c2
c3
z2
z1
c1
c4
z5
c6
c7
z4
c4
z6
c5
z7
c6
z6
z8
z9
c9
intensity
mass
0
50000
100000
150000
500
1000
1500
2000
2500
EAVKEESIK+XL_S
A_b_ion
A_y_ion
prec
b2+
y2+
b3+
y3+
y6++
y7++
y4+
y5+
b6+
y6+
y7+
b8+
intensity
m/z
0
500
1500
200
400
600
800
EAVKEESIK+XL_L
EHDDIFDKLK+XL_S
EHDDIFDKLK+XL_L
B_b_ion
B_y_ion
prec
y2+
b3+
y3+
y5+
y6+
y7+
y8+
b9+
intensity
m/z
0
2000
4000
6000
200
400
600
800
1000
1200
